# Relationship Between Resistin Levels and Sepsis Among Children Under 12 Years of Age: A Case Control Study

**DOI:** 10.3389/fped.2019.00355

**Published:** 2019-08-28

**Authors:** Lida Saboktakin, Nemat Bilan, Afshin Ghalehgolab Behbahan, Sadegh Poorebrahim

**Affiliations:** ^1^Pediatric Health Research Center, Department of Pediatrics, Tabriz University of Medical Sciences, Tabriz, Iran; ^2^Danesh Laboratory, Tabriz, Iran

**Keywords:** sepsis, resistin, pediatric, intensive care unit, pediatrics

## Abstract

**Objective:** The aim of this study was to investigate the level of resistin in children with and without sepsis hospitalized in the pediatric intensive care unit (PICU) and compare them to levels in healthy subjects in order to determine the trend of resistin levels in children in PICUs and also to identify the cut-off values for positive sepsis.

**Methods:** This was a case-control study conducted in 2014 at a children's hospital in Tabriz, Iran. Three groups were investigated, a case group comprised of patients with sepsis admitted to PICU and two control groups; one made up of patients admitted to PICU without sepsis and the other of healthy children. Variables included demographic, anthropometric (growth metric percentile), and clinical factors.

**Results:** Patients were randomized into control group A (*n* = 12, 48%), control group B (*n* = 11, 44%), and the sepsis group (*n* = 24, 47.1%). The difference in the means of resistin levels was significant on the first, fourth, and seventh days (*P* < 0.0001) in the case and control group A. Means comparisons in the case and control group B revealed significant differences on the fourth and seventh day (*P* = 0.005 and *P* < 0.0001, respectively) but not on the first day (*P* = 0.246). The trend of resistin levels increased in the septic group (F Huynh-Feldt = 37.83, *P* < 0.0001). The diagnostic accuracy of resistin level was high for discriminating sepsis (area under the receiver operating characteristic curve [AUC] 0.864 [SE = 0.41]). The sensitivity was 0.824 and specificity 0.72 with a cut-off point of 5.2 ng/ml on the first day.

**Conclusion:** In the present study, resistin level can be used as an indicator of sepsis in children admitted to PICU. However, the cut-off point based upon when a prediction could be made is different and is dependent on a variety of factors, such as control group and number of days since the first signs of sepsis.

## Introduction

Sepsis is defined as a life-threatening condition that is created by the body's chemical response to infection (1). It has remained a global healthcare concern (2) and a major cause of morbidity and mortality in PICUs (3–6). More than half of the deaths in children under-five occur from infectious diseases, such as pneumonia and diarrhea, which can lead to sepsis (7, 8). This imposes considerable financial costs on PICUs (4–6). More attention should be given to sepsis in developing countries where infectious diseases are life-threatening and healthcare resources are limited and insufficient (7–9).

The prevalence of sepsis-associated morbidity and mortality among children ranged from 5.6 to 27.3 and 12.5 to 24.0%, respectively (10–12). This rate, however, is not clear in Iran.

A global review of 26 countries, the frequency and mortality rate of severe sepsis was estimated to be 8.2 and 25%, respectively (13).

According to worldwide studies, there is a rising trend in the prevalence of pediatric sepsis (13–16). This may be related to high survival rates of patients at risk of developing sepsis, including those suffering from disorders associated with immunodeficiency or prematurity (4, 14). This high survival rate, however, does not detract from the importance of this issue.

Predictor variables can be helpful in prediction and management of sepsis. Resistin is a type of adipokine secreted by adipose tissue of mice (17). In human-based studies, resistin is expressed at very low levels in adipocytes and is produced by neutrophils and macrophages (18, 19). Resistin levels are also higher in patients with cardiovascular disease, atherosclerosis, rheumatoid arthritis, diabetes, obesity with insulin resistance, and sepsis (20–28).

It has been reported that resistin levels in patients with sepsis alter during an ICU stay (29, 30). This can be considered as a probable indicator of sepsis in neonates (1). Alterations in resistin levels in septic patients have been reported often (3), but the results are contradictory according to whether these levels rise or fall (3).

The aims of the present study were to: (1) compare the levels of resistin in the case group and the two control groups (healthy and PICU control groups); (2) evaluate the trend of resistin levels on the first, fourth, and seventh days; and (3) diagnose the accuracy of resistin level for discriminating sepsis in children.

## Materials and Methods

In this prospective study, a three-pronged strategy was used to compare groups. First was the inclusion of pediatric patients with sepsis admitted to PICU (case group) (50 cases). The second part involved pediatric patients without sepsis admitted to PICU (control group B) (22 cases) and the third included healthy children who had been referred to a health clinic for growth monitoring and had no clinical problems (control group A) (25 children). The first two groups (case and PICU control) included all patients who had been admitted to PICU from March to May 2014, while the third group included patients randomly selected from March to July 2014.

In this study, two control groups were specified due to the importance of the issue as well as the possibility of comparisons specific to each group. For example, duration of ventilation and length of PICU stay were variables that pertained to the PICU control group. This study was approved by the medical ethics committees of Tabriz University of Medical Sciences in April 2012.

Any children under 12 years of age admitted to PICU with sepsis were eligible for inclusion to the case group, any children under 12 years of age admitted to PICU without sepsis were eligible for inclusion to control group B, and any healthy children under 12 years of age referred to the health monitoring center were eligible for inclusion to control group A. Informed consent was obtained from the parents of all children included in this study. Exclusion criteria included: FBS > 400 mg/dl, failure to obtain a blood sample for resistin, genetic syndromes and major congenital anomalies, surgical conditions, cardiovascular dysfunction, and acute respiratory distress syndrome.

### Variables

Variables that were measured in case controls (case and control groups A and B) included demographic variables (age and gender), growth metric percentile, and resistin on the first, forth, and seventh days. Mechanical ventilation history, duration of ventilation, and length of PICU stay were variables that were compared only in the case and control group B.

### Growth Metric Percentile

In this study, a growth metric percentile based on gender, age, weight, and height, according to Centers for Disease Control and Prevention (CDC) growth calculator for 0 to 36 months and 2 to 20 years, was utilized (31, 32).

### Sepsis Determination

Sepsis was defined according to the criteria proposed by the American College of Chest Physicians/Society of Critical Care Medicine (33). Systemic inflammatory response syndrome (criteria for this disease met if at least two of the following are present: core temperature of >38.5°C or <36°C, abnormal heart rate, tachypnea, and abnormal leucocyte count) in the presence of infection detected by laboratory testing (CRP and WBC counts) was used to diagnose sepsis (34, 35).

### Statistical Analysis

Proportions regarding categorical variables and mean (standard deviation) or median (interquartile range [IQR]) for continuous variables were presented in deceptive statistics. Demographic differences within the groups were evaluated using the Mann-Whitney U test. Comparison between case and control groups based on resistin levels was performed using an independent sample *t*-test. The trend of resistin level was assayed by repeated measurement test. Accuracy was calculated using AUC. Statistical analysis was carried out using Predictive Analytics Software (PASW) statistics (version 18, SPSS Inc., Chicago, IL).

## Results

This study was conducted on 93 children between the ages of 1 month to 12 years. In this study, the sepsis group (50 cases) was compared with two control groups; a healthy control group (control group A) including healthy children who had been referred to a health clinic for growth monitoring and had no clinical problems (25 children) and a hospital control group (control group B) including pediatric patients without sepsis admitted to PICU (22 children).

Overall, there were 12 boys (48.0%) in control group A, 10 (45%) in control group B and 24 (48.0%) in case group (X^2^ in case and control group A = 0.006; *P* = 0.57, X^2^ in case and control group B = 0.063; *P* = 0.498 and X^2^ in control group A and control group B group = 0.081; *P* = 0.50). The age variable were significantly different in the case and control group B (*P* = 0.006). The baseline characteristics (gender, age, and growth metric percentile) of case group (*n* = 50), control group A (*n* = 25), and control group B (*n* = 22) are presented in Table 1.

**Table 1:** The baseline characteristics (gender, age, and growth metric percentile) of case group (*n* = 50), control group A (Healthy control group) (*n* = 25) and control group B (PICU control group) (*n* = 22).

**Variables**	**Median (IQ**		***P*-Value**	**Median (IQ**		***P*-Value**
	**Case**	**Control A**		**Case**	**Control B**	
Sex; Boy frequency (%)	24 (48.0)	12 (48.0)	0.006	24 (48.0)	10 (45.45)	0.498
Age; month**	6.0 (18.17)	2.0 (10.2)	0.058	6.0 (18.17)	18.0 (34.0)	<0.001
Growth metric percentile (%)**	45.0 (68)	43.0 (64)	0.96	45.0 (68)	42.5 (36)	0.677

^*^P-Value by Chi-square Test.

** *P-Value by Mann-Whitney Test*.

Of the 50 children, a 7-month-old infant deceased after 40 days of PICU stay; he was hospitalized for detection of pneumonia and had history of 240 h of ventilation.

The median (IQRs) of ventilation and PICU stay was 96 (100) h and 25 (19.75) days. These variables had significant differences in two groups of case and control group B (*P* = 0.011 and <0.0001, respectively). Resistin level on the seventh day had a positive correlation with PICU stay (*r* = 0.840) and mechanical ventilation (*r* = 0.657).

**Figure 1:**
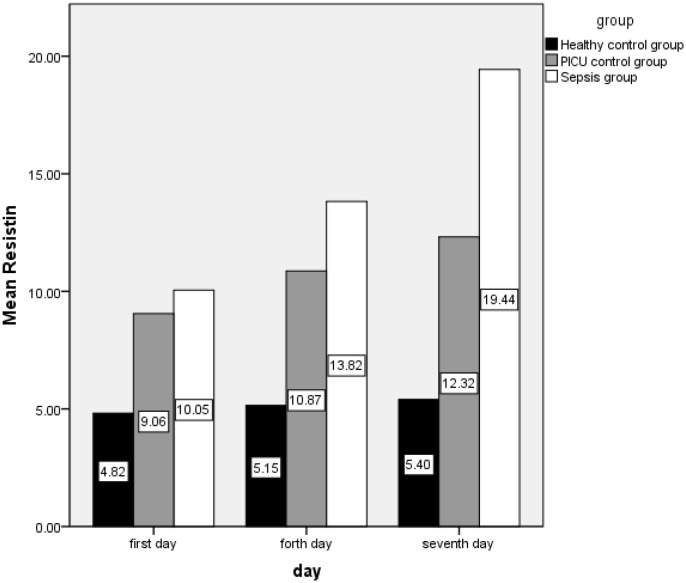
Distribution resistin level in first, fourth and seventh day by case, control A and B.

The mean (SD) of resistin(ng/m) in the first, fourth, and seventh days in the case group are as follows: 10.05 (0.46), 13.82 (5.62), and 19.44 (7.24), respectively, also this index was 4.82 (0.88) and 9.05 (2.72) on the first day, 5.15 (0.727) and 10.86 (3.24) on the fourth day, and 5.40 (0.66) and 12.32 (4.86) on the seventh day in control groups A and B, respectively (Figure 1).

The difference in resistin level means were statistically significant on case and control group A on the first day (*t* = −7.8, *P* < 0.0001), the fourth day (*t* = −10.82, *P* < 0.0001) and on the seventh day (*t* = −13.73, *P* < 0.0001). Comparison of means in the case and control group B was revealed to have significant differences on the fourth and seventh day (t_fourth day_ = −2.87, *P* = 0.005 and t_seventh day_ = −5.02, *P* < 0.0001) but not on the first day (t_first day_ = −1.17, *P* = 0.246). Comparison of the two control groups showed significant differences as well (t_first day_ = −7.24, *P* < 0.0001; t_fourth day_ = −8.44, *P* < 0.0001 and; t_seventh day_ = −6.91, *P* < 0.0001).

**Figure 2:**
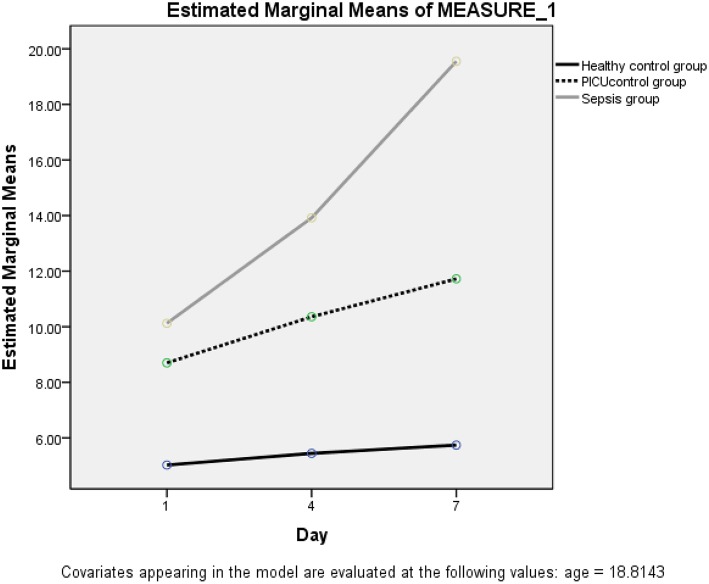
The trend of resistin serum concentration in case, control A and B.

The trend of resistin levels significantly increased after onset and admission to PICU in the septic (case) group. This result was earned by repeated measurement analysis in the presence of age and gender (F_Huynh−Feldt_ = 37.83, *P* < 0.0001) (Figure 2).

The AUC analysis was also calculated in order to estimate resistin levels on the first, fourth, and seventh day. The area under curve of sepsis level was 0.864 (SE = 0.41) for discriminating sepsis (sensitivity 82% and specificity 72% with a cut-off point of 5.2 ng/ml) on the first day. AUC of sepsis level on the fourth and seventh day were 0.987 (SE = 0.011) and 1.00 (SE = 0.0) respectively, with a cut-off point of 6.1 (sensitivity 98% and specificity 80%) and 7.5 (sensitivity 100% and specificity 100%). Findings of receiver operating characteristic analysis on case control group A and case control group B by age groups is accessible in Table 2.

**Table 2:** Distribution of area under the curve of resistin level, cut-off point, and diagnostic accuracy of sepsis by control groups, age and time.

**Variables**	**Groups**	**Cofactors**	**AUC (*P*-Value)**	**Cut off point**	**Sensitivity**	**Specificity**
Resistin in first day	Case- control A	Total	0.864 (<0.0001)	5.2	0.824	0.710
		1 month to 2 years	0.844 (<0.0001)	5.25	0.80	0.67
		2–12 years	0.909 (<0.0001)	5.1	0.90	1.00
	Case- control B	Total	0.555 (= 0.447)	–	–	–
		1 month to 2 years	0.987 (= 0.987)	–	–	–
		2–12 years	0.833 (= 0.039)	11.05	0.75	1.00
Resistin in fourth day	Case- control A	Total	0.987 (< 0.0001)	6.1	0.98	0.80
		1 month to 2 years	0.981 (< 0.0001)	6.3	0.925	0.952
		2–12 years	1.00 (< 0.0001)	6.17	1.00	1.00
	Case- control B	Total	0.664 (= 0.022)	11.35	0.627	0.88
		1 month to 2 years	0.61 (= 0.077)	–	–	–
		2–12 years	0.86 (= 0.011)	13.25	0.818	0.857
Resistin in seventh day	Case- control A	Total	1.00 (< 0.0001)	7.5	1.00	1.00
		1 month to 2 years	1.00 (< 0.0001)	7.5	1.00	1.00
		2–12 years	1.00 (< 0.0001)	9.2	1.00	1.00
	Case- control B	Total	0.760 (< 0.0001)	14.5	0.686	0.624
		1 month to 2 years	0.73 (= 0.001)	13.35	0.750	0.598
		2–12 years	0.916 (= 0.004)	17.2	0.909	0.857

## Discussion

The present study demonstrated that resistin levels on the first, fourth, and seventh day were statistically associated with sepsis. This outcome was obtained though analysis on septic pediatric subjects (case group) and healthy subjects (control group A). Resistin on the fourth and seventh day had a significant relationship with the PICU subjects with sepsis compared to the PICU subjects without sepsis (control group B).

According to the findings of a 2009 German study by Koch (37), resistin serum concentrations were significantly higher in sepsis than in non-sepsis patients hospitalized in ICU. Findings of another study by Aliefendioglu on premature newborns in NICU (38) was in line with the results of the present study. These results were consistent with findings of other studies by Macdonald et al. (1) and Galtrey et al. (2).

A main finding of this study was the different results gained from comparing the case group with control group B in comparison with the case group and control group A. We grouped the two control groups by their resistin levels and it was revealed that control B had significantly higher resistin levels than control group A. Children admitted to PICU are patients with certain conditions, and the concentration of resistin in them can be increased by various factors. Therefore, the difference between the two control groups in terms of resistin was expected.

Considering that some variables are PICU-specific, it was therefore essential to investigate a PICU control group in our research. In the present study, it was observed that the level of resistin increased during PICU stay. We also found that resistin levels increased If a patient was mechanically ventilated.” This result was consistent with findings of some other studies (1, 2, 39). Based on the studies, the main cause of acute respiratory distress syndrome was sepsis. In addition, sepsis increased susceptibility to ventilator-induced lung injury (40, 41); therefore, the relationship between sepsis with the length of stay in PICU and mechanical ventilation is two sided.

The cut-off point for septic children (case group) admitted to PICU (aged 1 month to 12 years) compared to healthy children (control group A) was estimated at 5.2 ng/ml based upon a sensitivity of 0.824 and specificity equal to 0.72 on the first day. The cut-off points had no difference after grouping by age (cut-off point for 1 month to 2 years old was equivalent to 5.25; 25 months to 12 years, 5.1). The cut-off point measure in case control B was higher than case control A.

In a study performed on newborn infants, a cut-off value of 8 ng/ml (sensitivity = 0.93 and specificity = 0.95) was calculated (42). Whereas, another study on premature newborns reported a cut-off value of 5.0 (sensitivity = 0.74 and specificity = 0.46) (18). The cut-off point of 6 (sensitivity = 0.90, specificity = 0.57) was calculated according to an investigation on 150 neonates admitted to King Abdulaziz University (36).

In terms of limitations of this study, it should be noted that the sample size was low, so it was not possible to conduct a multiple analysis.

## Conclusion

We found that certain levels of resistin on the first day can be used as a predictor of sepsis in neonates and children. However, the cut-off point based upon which a prediction can be made is controversial and dependent on a variety of factors including control group selection and time elapsed from the first signs of sepsis. This study demonstrated how the type of control group can affect the results.

## Data Availability

The raw data supporting the conclusions of this manuscript will be made available by the authors, without undue reservation, to any qualified researcher.

## Author Contributions

LS was the manager of project, and contributed to searching and analyzing. LS and NB contributed to clinical examinations. SP completed the paraclinical test. All authors contributed to the writing and editing of the manuscript.

### Conflict of Interest Statement

The authors declare that the research was conducted in the absence of any commercial or financial relationships that could be construed as a potential conflict of interest.
